# Emerging myxosporean parasites of Australian frogs take a ride with fresh fruit transport

**DOI:** 10.1186/1756-3305-5-208

**Published:** 2012-09-24

**Authors:** Ashlie Hartigan, Lee Peacock, Alex Rosenwax, David N Phalen, Jan Šlapeta

**Affiliations:** 1 Faculty of Veterinary Science, University of Sydney, Sydney, NSW, 2006, Australia; 2 Bird & Exotics Veterinarian, Green Square, Waterloo, NSW, 2017, Australia; 3 Present address: Institute of Parasitology, Biology Centre ASCR, Laboratory of Fish Protistology, Branišovská 31, České Budějovice, 370 05, Czech Republic

**Keywords:** Myxozoa, Cystodiscus, Translocation, Frog, Wildlife, Disease

## Abstract

**Background:**

The spread of wildlife pathogens into new geographical ranges or populations is a conservation concern for endangered species. *Cystodiscus australis* and *Cystodiscus axonis* are two species of myxosporean parasites infecting Australian frogs and tadpoles that have been recently recognised as important disease agents impacting amphibian conservation. Yet despite their importance to wildlife health, the mechanism of emergence for these parasites is unknown. We hypothesise that these parasites are capable of being accidentally translocated with their amphibian hosts in fresh produce (agricultural, horticultural and industrial) shipments into naïve environments and host populations.

**Methods:**

We surveyed 33 Australian “Banana box” frogs from Sydney fruit markets during 2011 using faecal smears and multiplex species specific PCR on DNA isolated from frog faeces or using histopathology to demonstrate the presence of both *C. australis* and *C. axonis*.

**Results:**

One of the “Banana box” frogs, the Dainty green tree frog (*Litoria gracilenta*) was positive for *C. australis* and *C. axonis* in its faeces and continuously shed the parasites for eight months.

**Conclusions:**

We present a possible mechanism for the emergence of *Cystodiscus* parasites and a non-invasive screening method to be used as a diagnostic test. In the future, vigilance and communication between wildlife managers/researchers and veterinarians will provide valuable information about these parasites, their host range and true distribution. This will aid risk management assessments for threatened populations within the range of *Cystodiscus* parasites and ultimately enhance conservation efforts.

## Background

*Cystodiscus australis* and *Cystodiscus axonis* are two species of myxosporean parasites infecting Australian frogs and tadpoles that have been recently recognised as important disease agents impacting amphibian conservation [1-3]. The genus *Cystodiscus* is globally distributed and may have ecological implications outside of Australia. Eight species in three frog families (*Litoria*, *Limnodynastes* and *Rhinella*) have been identified to be the hosts in New South Wales, Australia. Examination of archival specimens suggests that these parasites were not present in New South Wales prior to the mid 1960’s, but since then they have widely expanded their range and there is a significant prevalence of infection in several species of frogs [4]. How these parasites spread to New South Wales and where they came from is not known.

Thousands of frogs hitch rides every year in building materials, plants, and fresh food produce around Australia [5]. As an example, Sydney’s Flemington fresh produce market in New South Wales has been estimated to encounter at least 2,000 frogs annually [5]. Similarly, the markets of Melbourne in Victoria encounter between 6,000-8,000 frogs annually [6]. There are no other more rigorously derived figures or accounts of the numbers found in these markets, however, these numbers are probably an underestimation as many frogs will remain undetected, unreported, and can be lost on route to their final destination. In Australia, frogs that are found in fruit shipments are presented to herpetological groups and if they are considered to be fit and the species is appropriate for captivity the frog is rehomed. Routine practice is to quarantine frogs and then re-home them with an appropriate carer, never to be released back to the wild. However, no formal testing of translocated frogs is currently in place. In most cases, visual inspection of the frogs may be the only method used by the volunteers to determine their health status (authors personal observation). Therefore there is the possibility that pathogens carried in these frogs could enter their new environment if frogs escape detection. The precedence for this type of dissemination has already been set, by the anthropogenic world wide movement of Chytrid fungus (*Batrochochytrium dendrobatidis*) via environmentally resilient spores and/or frog trade [7,8]. Similarly, a lungworm *Rhabdias pseudosphaerocephala* was spread with the translocation of the Cane toad around the world [9] and a digenean *Haematoloechus floedae* was introduced to Costa Rica with *Rana catesbeiana *[10].

The aim of this study was to investigate if frogs translocated with fruit were a potential source of recently discovered emerging myxosporean parasites. To do this we surveyed frogs collected from Sydney fruit markets for evidence of *Cystodiscus* shedding in the faeces. An infected frog was found and it was shown to shed both species of *Cystodiscus* during the entire study period, supporting a possible means of dissemination of these parasites across Australia.

## Methods

### Frogs from fruit markets

In total 33 frogs were included in the study: White lipped tree frog, *Litoria infrafrenata* (n = 9); Green tree frog, *L. caerulea* (n = 6); Desert tree frog, *L. rubella* (n = 15); Peron’s tree frog, *L. peronii* (n = 2) and Dainty tree frog *L. gracilenta* (n = 1). All animals were found in fruit containers at Sydney markets, collected by the Frog and Tadpole Society and examined at the Bird and Exotics Veterinary Clinic (Greensquare, New South Wales). All animals were kept isolated in the clinic for faecal sample collection, before being re-homed for captive care.

### Histopathological examination

Tissue from two adult frogs *L. infrafrenata* was examined for the presence of parasites. The animals died whilst in care for unknown reasons. Tissues including brain, liver, gallbladder, kidney, spleen and stomach were fixed in 10% buffered formalin and processed for histological sectioning and staining with H&E as described previously [3].

### Qualitative and quantitative parasitological examination

Faecal samples were collected from all individually quarantined animals. Direct smear was used for both qualitative and quantitative examination. Faecal samples were weighed using balances with the precision of 0.001 g. Due to the small size of the faecal pellet (10–40 mg) no concentration techniques were employed. A tenth of the total volume of the faeces was mixed with 50 uL of Hartmann’s solution (Baxter Healthcare, Australia) and the sample viewed at 40x magnification (ISSCO BM-Lab microscope) and the entire slide observed for the presence of *Cystodiscus* spp. spores. During the parasitological examinations other suspected parasites were noted, including nematodes, ciliates and coccidian, but not reported in this study. Three smears were made for each sample and the number of *Cystodiscus* spp. spores counted and multiplied to enumerate the total number per faecal sample. Using the faecal weight the concentration was reported as spores per gram of faeces.

### DNA extraction and multiplex species specific PCR

DNA was extracted from frozen or ethanol (70-100%) fixed faeces from adult frogs using the FastDNA Soil Kit Protocol with a Fast Prep-24 homogenisation system equipped with QuickPrep Adapter (MP Bio, Australia). The speed setting used was 6.0 for 40s, otherwise the manufacturer’s instructions were followed as described previously [2]. Samples were eluted in kit buffer and stored at −20°C. PCR reactions were conducted in 25 μl volumes with MyTaq Red Mix (2X) (Bioline, Australia), 10 pmol each primer and 2 μl DNA (approx. 10 ng). Two PCR reactions were used (1) multiplex *C. axonis* and *C. australis* species specific PCR (primers S0116, S0119, S0110 and S0113) targeting ITS rDNA according to Hartigan *et al*. [3] and (2) myxosporea specific primers (primers MyxoSpecF and MyxoSpecR) targeting ~900 bp of the SSU rDNA according to Fiala [11]. Results were visualized on a 2% agarose gel stained with GelRed (Biotium, Australia). Positive samples were sent for sequencing at Macrogen Inc. (Seoul, South Korea) and analysed in CLC Main Workbench v6.2 (CLCbio, Denmark).

## Results

**Figure 1:**
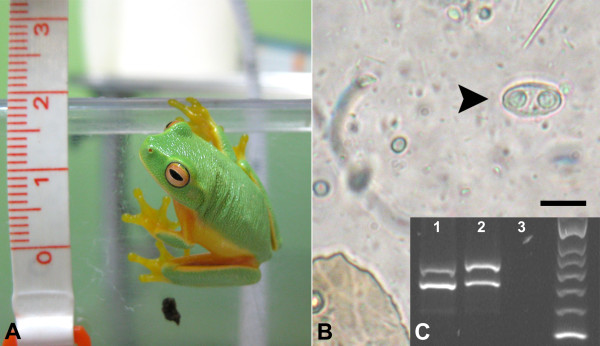
**A *Litoria gracilenta *, the Dainty tree frog infected with *Cystodiscus axonis *and *C. australis *. B *Cystodiscus *myxospore in faecal smear preparation, scale 10 μm. C** Multiplex species specific PCR result, 1- Positive control showing *C. axonis* (upper 597 bp band) and *C. australis* (lower 498 bp band), 2- faecal sample from *L. gracilenta* (positive for both *C. axonis* and *C. australis*) and 3- negative (water) control. Samples (5 μl) were run on 2% agarose gel, stained with Gel Red (Biotium, Australia) with Hyperladder II (Bioline, Australia) DNA ladder.

Faeces from 31 frogs were examined, a single Dainty GREEN tree frog (*L. gracilenta*) adult was found to be shedding *Cystodiscus* spores in its faeces (1/31, 3.23%) (Figure 1A). The individual appeared in good health throughout the study (September 2011 to April 2012), with an average faecal sample weight of 10-40 mg. All two-weekly parasitological examinations were positive for *Cystodiscus* spores (Figure 1B). Quantitative analysis of the faecal samples revealed an average of 416 myxospores per sample (average 30 mg) (range = 147–777, mean =417, st. dev = 139, CI 95% = 352- 481). Myxospores measured 14.8 (14–16) × 8.2 (7–9) μm (n = 30), had 5–10 transverse ridges on each valve, and the presence of caudal filaments was not clearly determined. The myxospore morphology overlapped those of *C. australis*, 16.0 (15–18) × 8.7 (8–10) μm with 5–11 transverse ridges, and *C. axonis*, 14.1 (13–15.5) × 8.5 (8–10.5) μm with 5–12 transverse ridges and caudal filaments.

Multiplex species specific PCR confirmed the presence of *C. axonis* and *C. australis* (Figure 1C).

No lesions (i.e. brain inflammation, liver fibrosis, biliary hyperplasia and/or myxozoan developmental stages) consistent with infection with either *Cystodiscus* spp were found in the brain, liver or gall bladder or in tissues from the two White lipped tree frogs (*L. infrafrenata*) that died spontaneously.

## Discussion

*Cystodiscus* parasites are associated with inflammation of nervous tissue and hepatic disease in a number of threatened and common frog species, yet the current distribution is still unknown [12]. It was demonstrated that up until the 1960’s frogs in Sydney were free of *Cystodiscus* parasites [4]. Their apparent emergence corresponds with improvements in roads and the increased use of refrigerated trucks, both of which could allow an increased survival of frogs that stowed away on transported material. For example, bananas have been cultivated in Australia since the 1870’s [13], with 90% of the bananas within Australia today produced in northern Queensland and distributed across the country. Large scale improvements to the transport of fresh produce around the country including the use of refrigerated trucks occurred in the second half of the last century [14]. We tested the hypothesis that frogs arriving in New South Wales in shipments of fruit and vegetables originating from other parts of Australia could be sources of *Cystodiscus* parasites.

Although only one infected frog was found in this small study set, its identification provides proof of concept that frogs from other geographic areas can disseminate both species of *Cystodiscus* to New South Wales and potentially other parts of Australia. The Dainty GREEN tree frog has a range that extends from northern New South Wales all the way to the northern tip of the east coast of Australia, it is a new host record for both *Cystodiscus* species. Thus it would be found in the fruit and vegetable growing areas of Queensland and therefore it is likely that this frog originated from outside of New South Wales. Our study shows that both parasites are shed in significant numbers in this translocated frog and that shedding can occur for a minimum of 8 months and thus this translocated frog and possibly others could be an important source of environmental contamination at their final destination.

Baseline data is essential for correct quarantine and health screens of any individual. Detection of myxosporean parasites can be extremely important especially if the infected host is a critically endangered species as observed in Australia’s most threatened frog, the Yellow spotted bell frog (*Litoria castanea*) infected with *Cystodiscus axonis*[15]. This study shows that both the simple non-invasive technique of faecal examination and a species specific PCR assay of faeces can be used to detect infected frogs and that both these techniques could be used in future studies to determine the full extent of *Cystodiscus* spp. movement in translocated frogs. Myxospores of myxosporean species are known to be resilient [16], so our data suggests that organizations rescuing frogs would benefit by being trained in reducing the risk of spread of *Cystodiscus* spp. and potentially other pathogens.

## Conclusion

We present a possible mechanism for the emergence of *Cystodiscus* parasites in New South Wales and Southern Queensland, Australia with an analogous mechanism for *Cystodiscus* parasites in other parts of the world where fresh produce is translocated across a large geographic distance. Vigilance and communication between wildlife managers/researchers and veterinarians provides valuable information about these parasites, their host range and true distribution. These findings aid risk management assessments for threatened populations within the range of *Cystodiscus* spp. parasites and ultimately enhance conservation efforts. Moreover, it emphasizes how the detection of translocated frogs and the ultimate disposition of these frogs should be considered by regulatory agencies to minimize the risk that they will spread new pathogens to naive frog populations.

## Competing interests

The authors declare that they have no competing interests.

## Authors’ contributions

All authors contributed to this study. AH, LP, AR, DNP and JŠ designed the study. LP and AH performed the experiments and collected data. AH and LP analysed the data. AH and JŠ drafted the manuscript. All authors read and approved the final manuscript.

## References

[B1] Murray K Skeratt L Marantelli G Berger L Hunter D Mahony M Hines H Guidelines for minimising disease risks associated with captive breeding, raising and restocking programs for Australian frogs. A report for the Australian Government Department of Sustainability, Environment, Water, Population and Communities A report for the Australian Government Department of Sustainability, Environment, Water, Population and Communities 2011

[B2] Hartigan A Fiala I Dyková I Jirků M Okimoto B Rose K Phalen DN Šlapeta J A suspected parasite spill-back of two novel Myxidium spp. (Myxosporea) causing disease in Australian endemic frogs found in the invasive cane toad PLoS One 2011 6 e18871 10.1371/journal.pone.0018871 21541340 PMC3081827

[B3] Hartigan A Fiala I Dyková I Rose K Phalen DN Šlapeta J New species of Myxosporea from frogs and resurrection of the genus Cystodiscus Lutz, 1889 for species with myxospores in gallbladders of amphibians Parasitology 2012 139 478 496 10.1017/S0031182011002149 22260881

[B4] Hartigan A Phalen DN Šlapeta J Museum material reveals a frog parasite emergence after the invasion of the cane toad in Australia Parasit Vectors 2010 3 50 10.1186/1756-3305-3-50 20537137 PMC2901343

[B5] O’Dwyer T Buttemer WA Priddel DM Inadvertent translocation of amphibians in the shipment of agricultural produce into New South Wales: its extent and conservation implications Pacific Cons Biol 2000 6 1 40 45

[B6] Centre AR The Lost frogs home: An initiative of the Victorian Frog Group to rescue accidentally relocated frogs In the spotlight. vol. 4 2003

[B7] Lips KR Diffendorfer J Mendelson JR Sears MW Riding the wave: Reconciling the roles of disease and climate change in amphibian declines PLoS Biol 2008 6 3 441 454 10.1371/journal.pbio.0060072 PMC2270328 18366257

[B8] Skerratt L Berger L Speare R Cashins S McDonald K Phillott A Hines H Kenyon N Spread of chytridiomycosis has caused the rapid global decline and extinction of frogs Ecohealth 2007 4 2 125 134 10.1007/s10393-007-0093-5

[B9] Dubey S Shine R Origin of the parasites of an invading species, the Australian cane toad (Bufo marinus): are the lungworms Australian or American? Mol Ecol 2008 17 20 4418 4424 10.1111/j.1365-294X.2008.03922.x 18803593

[B10] Brooks D Mclennan DA Leon-Regagnon V Hoberg E Phylogeny, ecological fitting and lung flukes: helping solve the problem of emerging infectious diseases Rev Mexicana Biodivers 2006 77 225 233

[B11] Fiala I The phylogeny of Myxosporea (Myxozoa) based on small subunit ribosomal RNA gene analysis Int J Parasitol 2006 36 14 1521 1534 10.1016/j.ijpara.2006.06.016 16904677

[B12] Hartigan A Dhand NK Rose K Šlapeta J Phalen DN Comparative pathology and ecological implications of two myxosporean parasites in native Australian frogs and the invasive Cane toad PLoS One 2012 7 10 e43780 10.1371/journal.pone.0043780 23056175 PMC3463585

[B13] Pearson M Tracking the Dragon: A guide for finding and assessing Chinese Australian heritage places 2002 Canberra: In. Edited by Commission AH

[B14] Scott KJ CSIRO Simple low cost ways to reduce wastage and extend the life of bananas after harvest 2010 Mudgee: Scott, K.J

[B15] Hartigan A Sangster C Rose K Phalen DN Šlapeta J Myxozoan parasite in brain of critically endangered frog Emerg Infect Dis 2012 18 693 695 10.3201/eid1804.111606 22469079 PMC3309702

[B16] El-Matbouli M Hoffmann RW Effects of freezing, aging, and passage through the alimentary canal of predatory animals on the viability of Myxobolus cerebralis Spores J Aquat Anim Hlth 1991 3 4 260 262 10.1577/1548-8667(1991)003<0260:EOFAAP>2.3.CO;2

